# Dissecting Through the Literature: A Review of the Critical Appraisal Process

**DOI:** 10.7759/cureus.59658

**Published:** 2024-05-04

**Authors:** Rawan Almutairi, Ahmad Alsarraf, Danah Alkandari, Hasan Ashkanani, Abeer Albazali

**Affiliations:** 1 Dermatology, Farwaniya Hospital, Farwaniya, KWT

**Keywords:** literature review of disease, literature review, review of literature, literature review, critical appraisal

## Abstract

Critical appraisal is a crucial step in evidence-based practice, enabling researchers to evaluate the credibility and applicability of research findings. Healthcare professionals are encouraged to cultivate critical appraisal skills to assess the trustworthiness and value of available evidence. This process involves scrutinizing key components of a research publication, understanding the strengths and weaknesses of the study, and assessing its relevance to a specific context. It is essential for researchers to become familiar with the core elements of a research article and utilize key questions and guidelines to rigorously assess a study. This paper aims to provide an overview of the critical appraisal process. By understanding the main points of critical appraisal, researchers can assess the quality, relevance, and reliability of articles, thereby enhancing the validity of their findings and decision-making processes.

## Introduction and background

Critical appraisal is a crucial process in evidence-based medicine, essential for rigorously evaluating research findings for their validity, relevance, and applicability to clinical practice. Various tools and checklists have been developed to guide the critical appraisal of different types of studies, including prediction modeling studies, outcomes in cancer patients using mobile health applications, and diagnostic accuracy studies [1-3]. Educators recognize the importance of training healthcare professionals in critical appraisal and biostatistics to enhance their ability to accurately interpret medical literature [4].

Healthcare professionals, including medical students and residents, are encouraged to cultivate critical appraisal skills to assess the trustworthiness and value of available evidence [5]. Research has shown that the lack of formal instruction in critical appraisal can hinder junior doctors' ability to effectively interpret clinical research [6]. Studies have underscored the significance of conducting critical appraisals of research papers and applying the findings to clinical practice [7].

In the context of evidence-based practice, critical appraisal is vital for determining which evidence should guide clinical decision-making and how to effectively apply evidence in practice [8]. Consumers of research often utilize critical appraisal tools to evaluate the quality of published research reports [9]. Moreover, the evidence-based medicine movement highlights the necessity of critically appraising randomized clinical trials to assess their validity, clinical impact, and patient applicability [10].

Although there are several materials accessible to assist with the process, there is no single "gold-standard" tool for critical appraisal. Therefore, this review provides an overview of the critical appraisal approach.

## Review

The critical appraisal starts by considering the following main points.

The relevance of the research question

Research questions play a crucial role in guiding research projects. They should be specific, feasible, and complex enough to warrant a detailed answer. Relevant research questions have a significant impact on clinical practice, improve outcomes, change protocols, and guide future research. Therefore, ensuring that the research question is relevant is essential to maintaining interest, focus, and clarity throughout the research process [11]. Additionally, the research questions should be clearly formulated and closely aligned with the study's goals, underscoring the importance of their relevance. Moreover, it has been explained that the research question should be broad enough to encompass all available research relevant to a particular topic or phenomenon of interest. This broader approach allows for a comprehensive evaluation of the existing literature to effectively address the research question [12].

Evaluation of the uniqueness of the research findings in relation to existing knowledge

When conducting a critical appraisal of a study, one key aspect to consider is whether the study adds anything new to the existing evidence in the field. This involves evaluating if the research question is relevant and if the study contributes novel insights or knowledge. The assessment typically includes inquiries such as "Does the study add anything new?" among others. This process helps researchers and professionals discern the value and originality of a study within the broader context of existing literature and knowledge [13].

Understanding different study designs

Any attempt to appraise the study's design with regard to its ability to infer cause and effect should center on evaluating whether it has avoided the various biases and confounding factors that can occur in observational studies, or between the treatment and control groups in an experiment. The consideration of these specific biases and confounding factors can be difficult, and it is often useful to have identified the most common threats to a study before attempting to appraise them [14].

This is because cause and effect relationships are inferred most reliably when strong designs that can provide an unambiguous answer are used, and it is an unfortunate fact that much clinical research attempts to address such relationships yet has used a weak study design. Weak designs are those where the possibility of an alternative explanation for the results is large, and in the worst case, it can be difficult to determine what is being observed. So-called “double-blind, placebo-controlled trials” are generally considered the strongest design for an experiment, and the equivalent “double-blind” trial is the best design for an observational study. In such studies, one can be more confident that an observed effect has not been influenced by external factors or the observer, and it is easier to establish what occurred during the study when it is later recounted or compared to similar investigations [15].

Once the design of the study has been established, it can be judged whether the most appropriate type of study has been used to address the research question. There are certain investigations for which a particular type of study is clearly the best method to address the research question, and in these cases, it is more useful to appraise whether the study design meets specific criteria for good research in that field. However, it is rare to find research that is not, by its nature, just an attempt to investigate a cause-and-effect relationship, and for these studies, it is generally best to assess whether the design is an experimental study or an observational study [16].

For example, clinical research may involve selecting a group of patients who will be followed up over time, in which case the research may be termed a cohort study. If the aim is to compare groups of patients with a certain characteristic, who are already receiving a particular treatment, to those who are not, it may be best to collect data using a case-control approach. An understanding of these different study designs provides a valuable initial framework with which to begin appraisal.

For example, although most research begins with a hypothesis, a particularly common error in clinical research is that the terms "experiment" and "observational study" are used interchangeably. This is confusing, as all experiments are observational in that they involve observing what happens when an intervention is made. The difference is that in an experimental study, the investigator intervenes to alter the natural course of events, whereas in an observational study, they observe events that occur in the absence of any interference. The way in which the data are collected can determine the study's design.

When critically appraising a research paper, it is essential that the study is interpreted and evaluated in the context of its design. Different study designs have different strengths and weaknesses, and these should be considered when assessing the validity and generalizability of the findings. It should not be assumed that one study is as good as another; this is not the case, and a poor study with a strong design could very well provide more reliable information than a strong study with a weak design.

Sources of bias addressed by methods

When critically appraising an article, there are important aspects to consider when evaluating methods to address sources of bias. Many questions should be asked when evaluating this section of an article. The type of bias should be identified, whether it is stated or not. Sources of bias are not always well documented, which can limit its assessment. A study may not want to admit it was biased for fear of losing credibility. In this case, an inference should be made based on the study design and analysis. It must consider whether each characteristic of a study is affecting internal or external validity so that a focus can be put on aspects most likely causing bias. As each source of bias is identified, the next step is to figure out if methods were taken to reduce this bias. Measures to reduce bias often result in strengthening the study in terms of validity and the ability to make causal inferences [17].

The source of bias that should be evaluated is confounding and the methods used to control it. A confounder is a variable that is associated with both an exposure and an outcome. It distorts or confuses the effect of an exposure on an outcome due to there being a common cause for the exposure and confounder or the confounder modifying the effect of the exposure. The study of confounding is typically a cause for bias, and a simple association between an exposure and outcome may be misconstrued as causal. Measures to address confounding in a randomized control trial may include restricting the study to specific criteria to eliminate potential confounding variables, matching subjects in the study, or stratification. Measures to address confounding in an observational study may include controlling for the confounding variable or multivariate statistical adjustment. By identifying whether confounding has been considered and how it has been controlled, we can determine the strength of a study regarding confounding and whether there is bias due to a confounded relationship between exposure and outcome [18].

An important element in evaluating methods to address bias is understanding it and the methods used. Understanding the bias and method will aid in determining whether it was effective in reducing bias and how much it did so. This will allow an overall judgment on the method to be made and whether the reader of the article can form an understanding of the effects of this bias and the study's attempt to remove it. Measures to remove bias are often complex and confusing, and it is easy for a study to claim that it has removed bias when it has, in fact, only reduced it or even increased it. Often, this area of study is poorly executed, and there is a need for a clearer explanation of complex methods so that readers can understand.

Deviation of the study from the original protocol

When considering the impact of deviation from the original study protocol, it is important to decide (in the case of negative results) whether the deviation has resulted in a type two error; this error is where a "false negative" is obtained, showing no difference between the intervention and control when there actually is a positive effect of the intervention. Type two error will occur if the deviation has resulted in a loss of power. This may have happened by changing an inclusion or exclusion criterion or the removal or change of the primary outcome measure. A common pitfall in power assessment is the assumption that because the sample size has been maintained, the power has also been maintained. In studies where a statistical test of power has not been documented, it is difficult to assess whether the sample size has remained sufficient in comparison to the new outcome measure. Types and directions of statistical analyses performed can be indicative of whether the power has been maintained [19-21].

The assessment of deviation impact should be framed around hypothesis testing. It is unlikely that this will be in the original statistical analysis plan but should be reconstructed as part of the critical appraisal of how the deviation may have influenced the results of the study. As with hypothesis testing in statistical analysis, the null hypothesis should be that the deviation from the original protocol has had no impact on the results of the study. The alternative hypothesis will differ depending on what is being tested but may be along the lines of "the deviation in the study has led to a change in outcome that supports the intervention."

Hypotheses identification

Prior to conducting a study, it is essential to identify and establish hypotheses derived from theory or previous experience. When a study examines the statistical significance of correlations that were not predetermined in the original hypothesis, there is a risk of obtaining false positive results. This is because, at a significance level of 5% (P = 0.05), one out of every 20 associations evaluated will be positive just by chance. When a substantial number of these tests are performed, false-positive findings may arise [22].

Identifying a hypothesis in a research article is crucial for several reasons. A research hypothesis, developed from a good research question, guides the objectives of the study. A well-formulated hypothesis also ensures that the study remains focused and purposeful throughout the research process. Moreover, hypotheses play a pivotal role in testing existing theories, providing a basis for empirical testing and validation. They help establish clear relationships between phenomena, paving the way for empirical testing and further investigation. Additionally, a hypothesis predicts the expected relationship and outcome, acting as a roadmap for the study and guiding data collection and analysis [23].

The appropriateness of the statistical analysis

Statistical analysis is fundamental in research, allowing researchers to derive meaningful insights from data. Ensuring the suitability of statistical methods used in a study involves aligning techniques with the study design, research question, and data characteristics. Key considerations for evaluating the adequacy of statistical analysis are as follows.

Match Between Study Design and Statistical Methods

Statistical methods should align with the study design [24]. Here are common study designs and their appropriate statistical tests.

Randomized controlled trial (RCT): This study design comprised a randomized controlled trial to compare the efficacy of two different topical treatments for psoriasis [25]. The appropriate statistical test for comparing the mean effectiveness of two treatment groups in an RCT is the independent samples t-test. This test compares the means of two independent groups to determine if there is a statistically significant difference between them regarding the outcome measure, such as reduction in psoriasis severity scores.

Case-control study: This study design comprised a case-control study investigating the association between sunscreen use and the risk of developing melanoma [26]. In a case-control study, where cases (individuals with melanoma) and controls (individuals without melanoma) are compared with respect to their exposure history (sunscreen use), the appropriate statistical test is logistic regression. Logistic regression allows researchers to estimate the odds ratio, which quantifies the association between the exposure (sunscreen use) and the outcome (melanoma) while adjusting for potential confounders, such as age, gender, and sun exposure.

Cross-sectional study: This study design comprised a cross-sectional study assessing the prevalence of contact dermatitis among healthcare workers [27]. For analyzing the association between exposure (e.g., occupational tasks) and outcome (contact dermatitis) in a cross-sectional study, the appropriate statistical test is the chi-square test of independence. This test evaluates whether there is a significant association between two categorical variables. In this case, it would assess whether there is a significant association between the type of healthcare tasks performed (exposure) and the presence of contact dermatitis (outcome) among healthcare workers.

Consideration of Data Distribution and Assumptions

Assess whether statistical assumptions, such as data normality, are met. If not, alternative techniques like non-parametric tests or variable transformations may be necessary to ensure accurate inference. For example, in a study on antipruritic cream effectiveness, assessing patient-reported itch score distribution informs the choice between parametric and non-parametric tests [28].

Handling of Missing Data

Missing data can introduce bias (attrition bias) if not addressed properly. Transparency in reporting missing data handling, whether through complete case analysis, imputation techniques, or sensitivity analyses, is essential for robust findings. In a longitudinal psoriasis biologic trial, transparent reporting of missing values ensures the integrity of results despite participant dropouts [29].

Multiplicity and Adjustments for Multiple Testing

Adjustments for multiplicity are vital when conducting multiple statistical tests or analyzing multiple outcomes to control type I errors and avoid false positives. Methods like Bonferroni correction or false discovery rate (FDR) control help maintain the integrity of findings. For example, in acne treatment comparisons, applying correction methods prevents false-positive results and ensures statistically significant findings are not due to chance alone [30].

Interpretation of Statistical Findings

Finally, critically evaluating how statistical findings are interpreted in the context of the study's research question and objectives is essential. Consider whether effect sizes, confidence intervals, and p-values are appropriately interpreted and whether the conclusions drawn from the analysis are supported by the data. By carefully evaluating the appropriateness of statistical analysis in a study, researchers can ensure the validity and reliability of their findings, ultimately enhancing the credibility and impact of their research.

Justification of the conclusion by the provided data

Conclusions are classically a summary of the study's intention, results, and discussion. Key findings are also mentioned in this part of the study, with possible justifications that can be added. Finally, a closing statement is added at the end of the conclusion with study recommendations [31].

Upon completing the data analysis, the results and discussion parts of the study are written using the data obtained. Data can include plain demographic and frequency data, as well as correlation data, photographic findings, and comparative analysis.

While study proposals and study ideas have anticipated results, the actual data, findings, and analysis results mandate and dictate the actual literature that must be written. Therefore, the conclusion of a study must be a summary of the provided results and analysis sections. Consequently, when appraising a paper for eligibility, power, and dependability, these factors must be considered. Checking the conclusion of a study must be a sequential process that follows the assessment of the results section

Conflict of interest

To critically appraise articles for conflicts of interest, it is crucial to consider the potential biases that may arise from financial relationships and industry affiliations. Studies have shown that conflicts of interest can impact research outcomes and the quality of publications. Disclosure of conflicts of interest is essential for transparency and maintaining the integrity of research [32]. A conflict of interest occurs when two or more contradictory interests relating to a study or a situation conflict. During a study, researchers need to take into account the conflicts of interest to ensure the safety of subjects. While reviewing the literature, it is crucial for any researcher to recognize and disclose any possible conflicts of interest that may influence the research selection and its legitimacy.

Consideration of ethical issues

Upon reviewing the literature, it is imperative to adhere to ethical considerations. Researchers have an obligation to present others' work faithfully, ensuring that quotations and the original intended meanings are maintained. Plagiarism is considered an academic offense; therefore, researchers should ensure that summarized works or paraphrases are in original wording. Additionally, it is important to ensure the credibility of cited material by using peer-reviewed articles and books. Furthermore, it is essential to maintain an unbiased and judgment-free mindset while working on and publishing research findings. This is achieved by seeking out all relevant literature, including studies with opposing viewpoints, as well as addressing gaps and acknowledging areas of contention.

Enhancing critical appraisal in healthcare through artificial intelligence

Incorporating artificial intelligence (AI) is progressively altering the framework of critical assessment in evidence-based practice. AI technologies, including machine learning algorithms and natural language processing tools, are being utilized in academia and research to enhance the examination of scientific publications [33].

These tools can swiftly identify patterns, assess statistical integrity, and even appraise the consistency of findings across similar studies. This trend not only speeds up the evaluation process but also enhances the accuracy and comprehensiveness of the assessments. Consequently, healthcare professionals can utilize AI to aid their decision-making, ensuring they are well-informed by accurately appraised and high-quality evidence [34].

The emergence of AI in healthcare also promises access to expert-level analysis, enabling professionals at all levels to engage with and apply the most recent research discoveries effectively. With AI's new automation trends for analyzing large datasets and providing insights into complex research medical data, healthcare professionals must use validation tools to ensure that their decisions are based on robust, accurate, and relevant information. This capability is crucial for maintaining high standards of care and patient safety in the rapidly evolving field of healthcare technology [35].

Table 1 shows a summary of the systematic process that involves assessing various key components in critically appraising an article based on the information from the search results.

**Table 1:** A summary of the systematic process for critically appraising an article [13].

Research Key Components	Description
Overview of the Paper	Check the publishing journal, year, article title, authors, and their institutions. Verify if there was a peer review process in journal acceptance protocols.
Abstract	Evaluate the clarity of the study's aim and materials/methods described. Look for a well-defined statement of questions focusing on participants, interventions, comparisons, outcomes, and study design (PICOS).
Methods	Examine study eligibility criteria, information sources, search strategy, study selection process, data extraction method, and risk of bias assessment.
Results	Review study selection details, characteristics, risk of bias within studies, meta-analysis results, and additional analysis methods.
Discussion	Analyze the main findings' summary, limitations discussion at the study and outcome level, and general interpretation of results in the context of other evidence.
Funding	Investigate the source and role of funders to identify any potential conflicts of interest.
Additional Considerations	Assess if the study question is relevant to your field and adds new evidence. Check if the study design matches the research question and addresses potential sources of bias. Ensure that the statistical analyses are performed correctly and that data justifies conclusions. Look for conflicts of interest and consider how the results can help in managing patients.

## Conclusions

Overall, critical appraisal plays a pivotal role in ensuring that healthcare professionals can make well-informed decisions based on high-quality, reliable, and relevant research evidence. By mastering critical appraisal skills, medical professionals can effectively navigate the extensive landscape of medical literature and apply the best available evidence to patient care.
